# Senescence- and Immunity-Related Changes in the Central Nervous System: A Comprehensive Review

**DOI:** 10.14336/AD.2024.0755

**Published:** 2025-08-26

**Authors:** Haiwen Feng, Junjin Li, Hongda Wang, Zhijian Wei, Shiqing Feng

**Affiliations:** ^1^Tianjin Key Laboratory of Spine and Spinal Cord, International Science and Technology Cooperation Base of Spinal Cord Injury, Department of Orthopedics, International Chinese Musculoskeletal Research Society Collaborating Center for Spinal Cord Injury, Tianjin Medical University General Hospital, Tianjin 300070, China.; ^2^Orthopedic Research Center of Shandong University and Department of Orthopedics, Qilu Hospital of Shandong University, Cheeloo College of Medicine, Shandong University, Jinan 250012, China.

**Keywords:** cell senescence, central nervous system, immunity

## Abstract

Senescence is a cellular state characterized by an irreversible halt in the cell cycle, accompanied by alterations in cell morphology, function, and secretion. Senescent cells release a plethora of inflammatory and growth factors, extracellular matrix proteins, and other bioactive substances, collectively known as the senescence-associated secretory phenotype (SASP). These excreted substances serve as crucial mediators of senescent tissues, while the secretion of SASP by senescent neurons and glial cells in the central nervous system modulates the activity of immune cells. Senescent immune cells also influence the physiological activities of various cells in the central nervous system. Further, the interaction between cellular senescence and immune regulation collectively affects the physiological and pathological processes of the central nervous system. Herein, we explore the role of senescence in the physiological and pathological processes underlying embryonic development, aging, degeneration, and injury of the central nervous system, through the immune response. Further, we elucidate the role of senescence in the physiological and pathological processes of the central nervous system, proposing a new theoretical foundation for treating central nervous system diseases.

## 1. Introduction

Senescent cells were initially identified and defined by Hayflick and Moorhead, who introduced the concept of replicative senescence following their observation of the limited proliferative capacity of primary human fibroblasts in vitro. Replicative senescence is characterized by telomere shortening in normal cells due to continuous division. Upon reaching a critical telomere length, cells autonomously initiate the aging process [1]. Subsequent investigations of cell senescence have shown that the factors contributing to this phenomenon include oncogene activation, chemotherapy drug usage, oxidative stress, viral infection, epigenetic alterations, DNA damage, mitochondrial dysfunction, metabolic changes, and ongoing cellular division [2-8].

Senescent cells exhibit distinct differences from normal cells in terms of their morphology and molecular markers. Compared with normal cells, senescent cells display an increased volume and flatter shape [9], in addition to a notable upregulation of β-galactosidase (SA-β-gal) due to heightened lysosomal hydrolysis activity and the accumulation of cytoplasmic granules [10]. Furthermore, activation of cell cycle inhibitory proteins, such as P16INK4a, P21CIP1, and P53 [11-13], along with reduced lamin B1 levels resulting from nuclear lamina disruption and increased heterochromatin lesions due to DNA damage, have also been observed [14-16]. Additionally, senescent cells secrete a plethora of active proteins, including inflammatory cytokines, growth factors, and extracellular matrix proteins, collectively referred to as the senescence-associated secretory phenotype (SASP), which exerts paracrine effects on surrounding tissues [17-19].

Senescent cells represent a complex phenomenon; their impact is contingent upon the timing and location of their emergence, as well as their interaction with the surrounding microenvironment [20]. For example, the transient occurrence of senescent cells during embryonic development and tissue repair can confer advantageous effects [21]. During embryonic development, senescent cells have been observed to serve as signals for immune cells to facilitate the formation of specific organs [22]. For example, during kidney development, senescent cells play a crucial role in promoting mesonephrotic degeneration by signaling macrophages and facilitating the propagation of the signals necessary for normal organ structure formation [23]. Furthermore, during the tissue repair process, senescent cells may reduce fibrosis and enhance wound healing, as evidenced by skin and corneal wound healing. Aging fibroblasts have also been shown to mitigate fibrosis, thereby promoting tissue repair [24, 25]. However, long-term accumulation of senescent cells can have detrimental effects, as they may induce a pro-inflammatory microenvironment through the continuous release of age-associated secretions, thereby inducing the creation of the senescence-associated secretory phenotype (SASP), which promotes tumorigenesis. For example, in ovarian and liver tumors, the presence of senescent cells can trigger a pro-inflammatory response that facilitates tumor cell proliferation [26, 27]. Similarly, senescent stem cells may contribute to cartilage degradation in bones and joints by secreting factors, such as DDK1, which lead to the development of arthritis [28].

**Figure 1.**
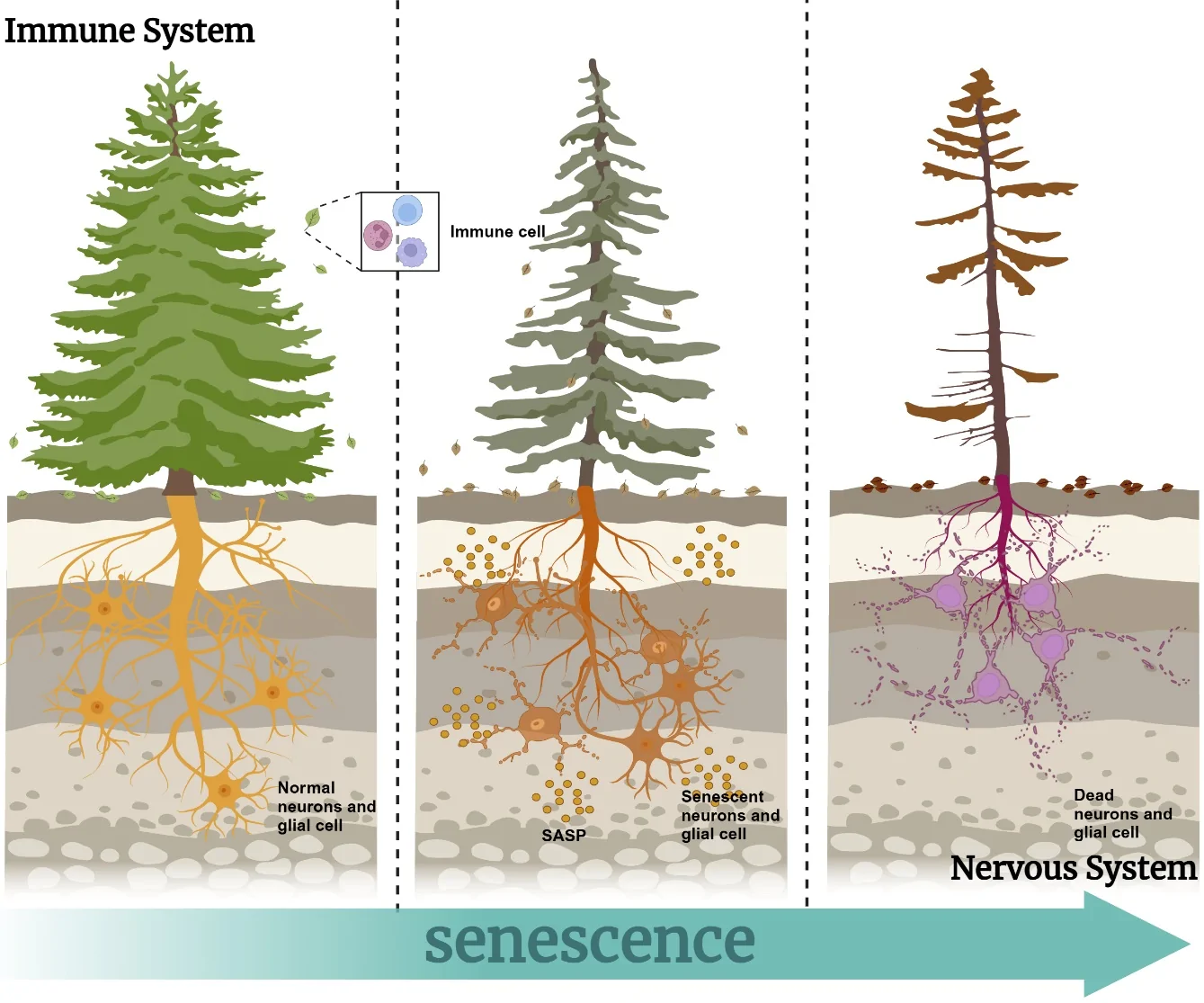
**The role of cellular aging in the interaction of the CNS and the immune system**. The leaves represent immune cells in the immune system, and the roots represent nerve cells and glial cells in the central nervous system. The aging of the immune system and the central nervous system contribute to the aging of the other. Understanding the role of cellular aging in the immune system and the central nervous system will help to advance the research of central nervous system diseases. Created with BioRender.com.

Both the central nervous and immune systems play pivotal roles in the human body, and their interactions are indispensable for maintaining overall health. Over time, cells in both systems undergo aging, in processes which, rather than being isolated, mutually exacerbate each other. Cellular senescence in the central nervous system not only affects the function of the central nervous system itself, but also directly influences the immune system via a series of mechanisms. This function is primarily mediated by the SASP, which is secreted by senescent cells in the central nervous system. The SASP contributes significantly to the activation, migration, adhesion, and differentiation of immune cells, thereby influencing the overall function of the immune system. The presence of senescent cells can further alter the distribution of immune cells in tissues, thereby affecting normal immune system function. Cellular senescence in the immune system can also lead to a cascade of effects. Aged immune cells commonly experience a decline in their ability to effectively respond to harmful cells or molecules within the body, thereby resulting in a diminished capacity to recognize and eliminate pathogens, increasing the susceptibility to infection and disease [29-31]. Furthermore, aging immune cells may expedite the aging process in other organs through a series of signaling molecules, consequently promoting systemic aging (Fig. 1) [29, 32]. Hence, understanding the intricate interplay between the central nervous and immune systems, along with their respective aging mechanisms, is important for the prevention and treatment of age-related diseases. However, further investigations are warranted to delve deeply into the complex interactions between these systems and devise targeted interventions aimed at delaying the aging process and preserving overall health.

**Figure 2.**
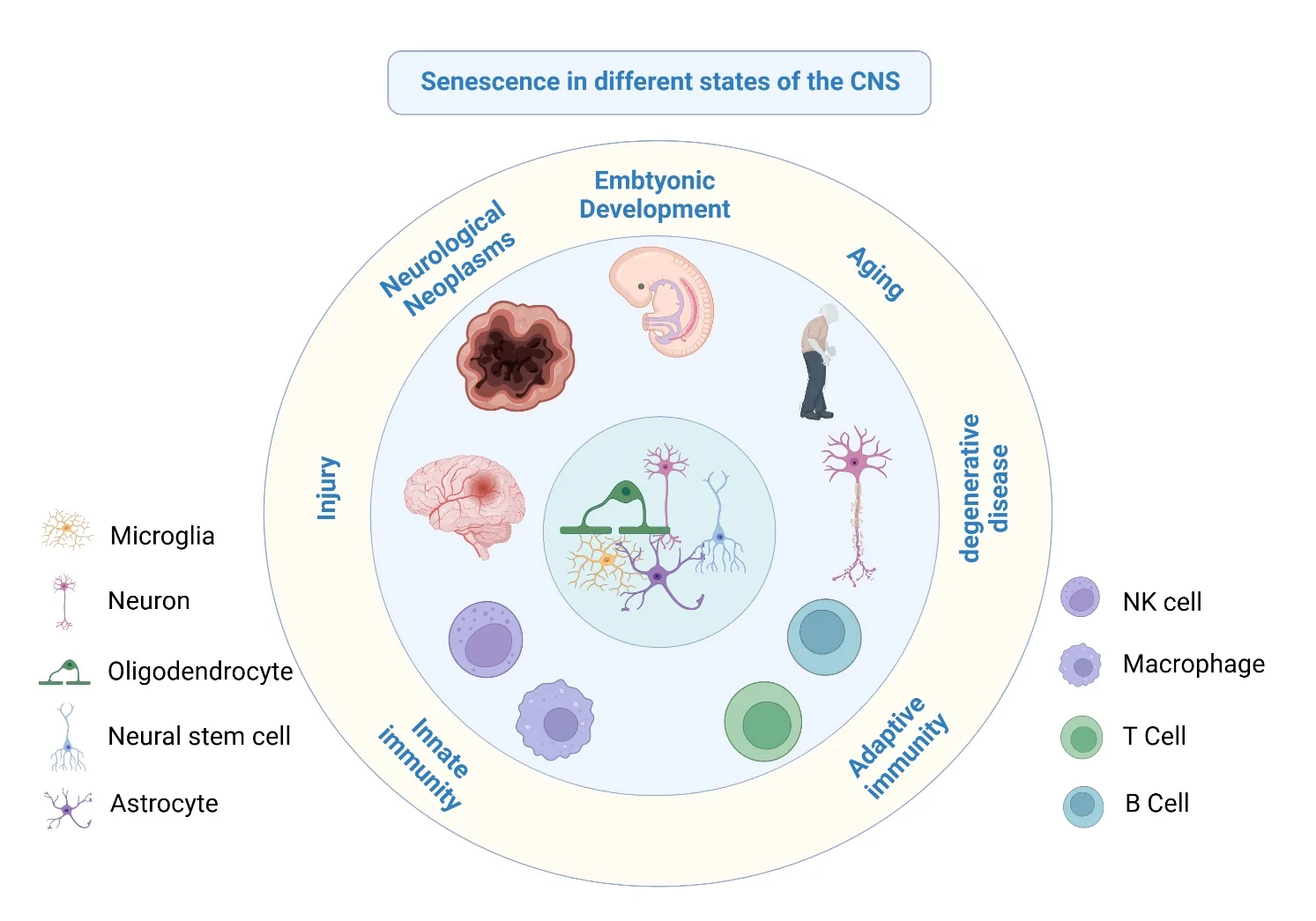
**Senescence in different states of the CNS**. The role of different cellular senescence, including neurons, neural stem cells, microglia, astrocytes, and oligodendrocytes, in embryonic development, aging, degeneration, injury, and neurotumor in the central nervous system is discussed in detail here. At the same time, the role of immune cell senescence in innate and adaptive immunity, including NK cell, macrophage, T cell and B cell, was discussed. Created with BioRender.com.

In this context, the present review aims to explore how senescence contributes to the physiological and pathological processes underlying embryonic development, aging, degenerative diseases, and central nervous system damage through the modulation of immune responses (Fig. 2).

## 2. Embryonic Development

Senescence is a fundamental component of embryonic development, characterized by a conserved and temporally-specific programmed process restricted to distinct structural regions [23]. Senescent cells have been identified in various embryonic tissues, including the mesonephric tubules, endolymphatic sac, apical ectodermal ridge, interdigital web, and neural tubes [22, 33]. These senescent cells exhibit positive staining for SA-β-gal activity, increased expression of cell cycle inhibitor proteins (p15, p21, p27), and negative staining for Ki67 and BrdU. Notably, the upregulation of P21 has been shown to play a pivotal role in this process through its association with the activation of TGF-β/SMAD and PI3K pathways. However, no increase in P16 expression or DNA damage has been observed during embryonic development [22, 23]. This distinction indicates that aging during embryonic development differs from age-related diseases or injuries, in which increased P16 expression and DNA damage are both key factors. This indicates that cellular senescence plays distinct roles in embryonic development, age-related diseases, and injuries.

Macrophages play an important role in the elimination of senescent cells during embryonic development. Examination of the developmental stages of mouse embryos has shown that the mesonephric tubules undergo senescence and subsequent degeneration as part of their normal development. At E14.5, senescent cells release TGF-β to recruit a large number of macrophages to infiltrate into the mesonephric tubules, leading to their elimination by E15.5. In P21 knockout mice, minimal macrophage infiltration was observed at E14.5, while the mesonephric tubules remained intact at E15.5; however, they were eventually eliminated through a delayed apoptotic program involving the recruitment of macrophages at E16.5. Furthermore, macrophage recruitment by senescent cells also occurred in the apical ectodermal ridge (AER), where a significant number of senescent cells were present in the ectoderm on days E11.5-12.5, followed by an influx of macrophages around these cells on days E13.5-14.5, prior to the gradual disappearance of senescent cells. Unlike the mesonephric tubules and AER, the ES does not undergo elimination. During the development of the endolymphatic sac, knockout of P21 results in abnormal expansion of pendrin-negative cells following E14.5, which may be linked to the loss of senescence [22, 23].

The closure of the neural tube during embryonic development is crucial for brain and spinal cord formation [34]. Neural tube defects resulting from the failure of this closure are the second most common structural birth abnormalities globally. The etiology of these defects is intricate, involving a complex interplay between both genetic and environmental factors [35]. Adequate maternal nutrition is essential for proper neural tube closure; as such, maternal metabolic disorders may pose a significant risk for neural tube defects. Embryos of maternal mice with gestational diabetes mellitus exhibit premature senescence of neuroepithelial cells, concomitant with an increase in the occurrence of neural tube defects. Maternal diabetes during pregnancy can induce premature senescence of neuroepithelial cells in the embryo, thereby increasing the risk of neural tube defects. Following a series of gene knockout tests, the premature senescence of neuroepithelial cells was found to be mediated by the FoxO3a-Mir-200c-ZEB1/2-p21/p27 pathway. Knockout of FoxO3a, miR-200c, p21, or p27 effectively inhibited embryonic senescence in diabetic maternal mice and reduced the risk of neural tube defects. The severity of developmental abnormalities has also been shown to be correlated with the extent of neuroepithelial cell aging. Although moderate aging may not manifest as neural tube defects, it can lead to conditions such as microcephaly, autism, and other developmental disorders, whereas severe aging may trigger neural tube defects. Furthermore, rapamycin has been shown to suppress embryonic neuroepithelial cell senescence and decrease the incidence of neural tube defects in maternal diabetic mice by inhibiting the mTOR pathway [36].

Senescence is widely observed during embryonic development, and the regulation of embryonic development by normal aging plays a pivotal role in this process. Indeed, cellular senescence is indispensable for embryonic development as it contributes to organ differentiation during this crucial stage. However, unexpected cell senescence can impede normal progression of embryonic development, affect the formation of healthy tissues and organs, and trigger functional impairment. Together, this evidence suggests that the macrophage-mediated clearance of senescent cells is crucial to maintain their normal function.

## 3. Aging

As individuals age, a range of cells inevitably experience damage as a function of their normal physiological processes, thereby contributing to the aging process. As damage continues, senescent cells continue to accumulate in diverse tissues and organs, leading to a functional decline in normal tissues [37]. The relationship between aging and cellular senescence has been demonstrated in prematurely aging mice with knockdown of BubR1, a spindle checkpoint gene that oversees correct chromosome segregation during mitosis. These knockdown mice exhibit an increased proportion of senescent cells, and display various aging-associated phenotypes, including sarcopenia, cachexia, cataracts, and lordosis [38-41]. Furthermore, BubR1 knockdown mice show a significant increase in P16ink4a positive cells. Subsequent specific knockdown of P16ink4a resulted in decreased expression of aging markers, reduced presence of senescent cells, and mitigation or delay of the development of age-related phenotypes [39]. Biochemical changes following cellular aging include abnormal protein accumulation, metabolic alterations, autophagy dysfunction, mitochondrial dysfunction, and oxidative stress, all of which can contribute to neurodegeneration and impaired cognitive function [42-46].

### 3.1 The aging immune system

Multiple longitudinal analyses of the elderly immune system have revealed distinct differences in immune balance between young and old individuals. As individuals age, their immune cells undergo gradual changes, resulting in shifts in immune cell composition at varying rates. This ultimately results in the transformation of the aging immune system into a new state of immunological equilibrium in older individuals [47]. The primary indications of immune system aging include diminished lymphocyte production and compromised adaptive immunity [48]. Senescence of hematopoietic stem cells is a fundamental cause of immune senescence, which directly affects the generation and functionality of B and T cells [49]. In young bone marrow, hematopoietic stem cells maintain a balance between lymphocyte output and bone marrow cell production, whereas in aged bone marrow, the output of bone marrow cells dominates, leading to reduced adaptive immunity supported by lymphocytes, and an increase in the inflammatory response in vivo [49, 50]. Furthermore, elderly individuals exhibit decreased initial T cells in their blood, along with an increase in T and B cells expressing more age-related genes that can affect memory immune cell function [51, 52].

### 3.2 Aging brain

As the body ages, the proportion of senescent cells in the brain increases, leading to a decline in normal brain functions, including memory, spatial perception, and learning ability. Impairment of brain function by aging cells is commonly accompanied by immune cell activity. Dendritic and T cells function as key components of the inflammation that occurs during brain aging. In the normal adult brain, parenchymal dendritic and T cells are scarce, and limited to the dura and choroid plexus; however, the aging brain shows an increase in dendritic and T cells within the brain parenchyma [53]. In aged mice, CD8+T cells predominantly accumulate in the subventricular zone (SVZ) of the brain, in contrast to a decline in T cells in the aging bloodstream. This indicates that chemokines released by microglia, endothelial cells, or macrophages in the aged brain serve as antigens that activate CD8+T cells in the SVZ. These activated T cells express interferon-γ and have been shown to inhibit both the in vitro and in vivo proliferation of neural stem cells, ultimately impairing neurogenesis and cognitive function [54]. Further, the disruption of normal meningeal T-cell trafficking through the surgical removal of deep cervical lymph nodes results in a significant increase in CD4+ T-cells within mouse meninges, accompanied by a notable impairment in spatial learning and memory among subjects in the resection group [55]. In immune-compromised adult mice, the lack of specific T cells in the hippocampus severely impairs neurogenesis. However, T-cell supplementation can restore and enhance hippocampal neurogenesis, thereby improving spatial learning and memory in mice [56]. These findings further underscore the intricate impact of T-cell accumulation on brain aging, thus highlighting the need for more detailed investigations into the effects of different T-cell activities on the central nervous system. Additionally, one separate study revealed that cellular senescence predominantly occurs in neuroblasts within the dentate gyrus during human brain aging [57]; indeed, the dentate gyrus is the primary site of neurogenesis in humans, and this process thus significantly impacts human memory and cognitive function [58, 59]. Furthermore, aging neuroblasts have been shown to promote the expansion of NK cells within the dentate gyrus, in addition to augmenting their cytotoxicity via IL-27 secretion. Consequently, this leads to a disproportionate increase in NK cells compared to other immune cell subsets during aging, which ultimately affects neurogenesis and cognitive function. Overall, these results suggest that NK cells play a crucial role in immune surveillance during brain aging by influencing neurogenesis and cognition [57].

### 3.3 Aging of the spinal cord

During aging, the proportion of senescent cells within the spinal cord increases, subsequently influencing its normal functionality and increasing its susceptibility to spinal cord diseases [60]. The spinal cord further serves as a crucial conduit, linking the brain to peripheral nerves [61]. The coordination capacity of autonomic nerves within the spinal cord is intricately associated with cardiovascular, respiratory, digestive, urinary, and reproductive functions [62]. Aging of the cardiac autonomic nervous system results in the downregulation of miR-145 and disinhibition of the neural rejection factor Semaphorin-3A, leading to reduced nerve density in the left ventricle, heightened arrhythmia incidence, and a diminished heart rate variability. The administration of senolytics to eliminate senescent cells may increase nerve density, thus enhancing cardiac function [63]. The interplay between nerves and immune cells regulates vascular inflammation [64]; as such, further exploration of the involvement of immune cells in the neuroregulation of cardiac aging is required. Indeed, one study involving both young and aged dogs revealed that older dogs exhibit a higher proportion of activated microglia localized around the motor neurons in the lumbar spinal cord. This age-related regional variation in microglial activation has been implicated in rendering lumbar motor neurons more susceptible to age-related pathological changes [65]. Subsequent immunostaining of the spinal cord in experiment using both humans and non-human primates revealed an increase in senescent cells within the aging spinal cord, particularly affecting motor neurons located in the ventral grey matter, a phenomenon which was attributed to the presence of CHIT1 + microglia driving motor neuron senescence. This particular subtype of microglia aggregates around the motor neurons and releases CHIT1 to induce activation of the TGFβ1-SMAD2 pathway in the motor neurons, thus to cellular senescence. While CHIT1+ microglia were not observed in mice, the administration of exogenous CHIT1 resulted in the induction of motor neuron senescence in the mouse spinal cord, thereby indicating sustained cellular senescence triggered by activation of the TGFβ1-SMAD2 pathway. Furthermore, studies have consistently demonstrated that the injection of vitamin C effectively reduces neuronal aging, an effect which can be attributed to its potent antioxidant effects. This indicates the significant involvement of oxidative stress in spinal cord neuronal aging [66].

Aging of the nervous system has also been shown to be positively associated with neuronal and glial cell senescence, which could potentially be attributed to the DNA damage induced by oxidative stress. As the central nervous system undergoes ages, corresponding changes are observed in the distribution and activity of immune cells throughout the body. As such, the crucial role of immune cell activity in maintaining central nervous system function cannot be overstated. Beneficial immune cell activities further play a key role in eliminating senescent cells or mitigating their detrimental effects on the central nervous system, whereas adverse immune cell activities can exacerbate damage and impair neurogenesis and neural function.

**Table 1:** **The effect of different senescent cells**. Here we show the effect of senescence of different cells in the central nervous system or immune system on the function of the central nervous system and the promotion of degeneration.

System	Cell	Feature	Function	Disease	Research type
CNS	Neuron	SASP release increases	promotion of axonal loss and synaptic disconnection	Alzheimer's disease	Animal experiment
	Astrocyte	exhibit proinflammatory phenotypes	Impaired support and phagocytosis , promoting dopaminergic neuron death	Parkinson's Disease	Animal experiment
	Microglia	lysosomal accumulation, telomere shortening	compromised ability to phagocytose Aβ protein	Alzheimer's disease	Animal experiment
Immune system	PBM	aging-related changes	Cerebrospinal fluid mobility is reduced and waste removal is impeded	Alzheimer's disease	Animal experiment
	T cell	Telomere length is shortened	The release of proinflammatory factors increased	Alzheimer's disease	Clinical research
	CD8+T cell	Telomere length is shortened	Proinflammatory factors increase, hindering myelin regeneration	Multiple sclerosis	Clinical research

## 4. Degenerative diseases of the central nervous system

Age-related neurodegenerative diseases exert a substantial impact on the health of elderly individuals [67], with conditions such as Alzheimer's disease [68], Parkinson's disease [69], and amyotrophic lateral sclerosis [70] all associated with premature mortality in animal models. In humans, these diseases are predominantly observed in the elderly, and contribute to a reduced lifespan, emphasizing the close association between age and degenerative diseases of the central nervous system [67]. Aging of the central nervous system is also accompanied by physiological changes in various cell types within the system, which underlie the pathological mechanisms driving the onset of these degenerative diseases. For example, one study using a rat model of facial nerve avulsion showed that aged rats exhibited heightened vulnerability and diminished resilience to neuronal injury [71]. In vitro experiments with astrocytes have further demonstrated no apparent aging phenotype as telomeres alternately shorten and lengthen [72]. However, in vivo studies have shown that astrocytes exhibit signs of aging, particularly through increased expression of GFAP and vimentin filaments; elevated secretion of IL-6, IL-β, and TNF-α; as well as ultrastructural changes in the nucleus and accumulation of lipofuscin in the cytoplasm, which contribute to brain dysfunction [72]. Both in vivo and in vitro studies have shown that microglia display aging characteristics, including impaired migration and phagocytosis, leading to toxicity and inflammatory overreactions in the central nervous system [73]. Similarly, oligodendrocytes undergo senescence both in vivo and in vitro. Senescent oligodendrocytes display enlarged and flattened cell morphology, exit the cell cycle, and accumulate lysosomes in the cytoplasm, all of which have detrimental effects on myelin generation [74]. The infiltration of lymphocytes into the central nervous system can directly modulate environmental homeostasis. The proportion of senescent CD8+T cells increase with age, leading to an increase in the production of inflammatory mediators, as well as the promotion of central nervous system inflammation. Increased inflammation is associated with cognitive dysfunction, including impaired information processing, working memory, and spatial memory [75] (Table 1).

### 4.1 Alzheimer's disease

Alzheimer's disease is a highly age-correlated neurodegenerative condition, characterized by the high expression of brain cell proteins P16 and P53, indicating the involvement of senescent cells in its pathogenesis. This cellular aging process leads to an increase in the neuronal population, heightened nerve inflammation, and the promotion of axonal loss and synaptic disconnection [76, 77]. A higher proportion of significantly senescent neurons has further been observed in the prefrontal cortex of patients with AD, thus demonstrating a bystander effect. Senescent neurons can elevate inflammatory components in the cerebrospinal fluid by inducing SASP, thereby triggering astrogliosis. Pharmacological depletion of senescent neurons has been shown to mitigate inflammation in both the brain and cerebrospinal fluid and impede the decline of cognitive function. These findings indicate that the targeted elimination of senescent neurons may represent a promising strategy for the treatment or prevention of AD [78]. Furthermore, the pathogenesis of AD is closely linked to the activation of microglia, leading to the upregulation of inflammatory mediators in the brain, which is consistent with observations in Alzheimer's patients [79-81]. AD is accompanied by persistent and robust microglial proliferation; however, failure to effectively clear β-amyloid and downregulate excessive inflammatory responses contribute to inflammation-mediated cytotoxicity and cognitive impairment [82]. Early microglial proliferation can impede aging-induced progression of Alzheimer's disease, reduce Aβ protein accumulation, and mitigate neuroinflammation. Conversely, excessive proliferation can induce replicative senescence in microglia, characterized by lysosomal accumulation, telomere shortening, heightened expression of senescent-related genes, and compromised ability to phagocytose Aβ protein—exacerbating neuroinflammation and synaptic damage [83]. In one study, senescent microglia were induced by PLX3397 in young mice through multiple in vivo microglial depletions; these experiments demonstrated that the senescent microglia themselves can contribute to cognitive decline, and that their impact on brain function is mediated through communication with other cells in the brain [73]. The in vitro culture of microglia isolated from young and aged mice revealed that microglia from aged mice exhibited increased secretion of inflammatory factors, reduced responsiveness to stimulation, and a diminished ability to phagocytose Aβ proteins, thereby promoting amyloid deposition in the aging brain, and facilitating the development of AD [84]. The group of perivascular and leptomeningeal macrophages, collectively referred to as parenchymal border macrophages (PBM), are located near the central nervous system, and play a crucial role in regulating cerebrospinal fluid flow. The injection of macrophage colony-stimulating factor (M-CSF) within the brain pool can lead to aging-related changes in PBM appearance, resulting in decreased cerebrospinal fluid liquidity and impaired waste removal, thereby promoting Alzheimer's disease [85]. The analysis of telomere length in immune cell populations associated with Alzheimer's further revealed a correlation between T-cell telomere length and AD onset and progression. Furthermore, shortened telomeres in T cells have been linked to increased serum levels of pro-inflammatory factors, suggesting that senescent T cells may contribute to the development of AD [86]. The present study demonstrated the involvement of diverse cellular senescence mechanisms in the pathogenesis of Alzheimer's disease, suggesting that a comprehensive approach targeting cellular senescence should be considered for the treatment of this disorder.

### 4.2 Parkinson's Disease

The core pathological features of Parkinson's disease (PD) include the early death of substantia nigra neurons and motor dysfunction caused by the lack of dopamine in the basal ganglia due to α-synuclein misfolding [87]. The prevalence and incidence of PD increase with age, and senescent cells have been detected in the dead tissues of PD patients, indicating a potential relationship between PD and aging [88, 89]. Astrocytes typically provide support to neurons through the secretion of neurotrophic factors [90]; however, in animal studies, exposure to certain environmental factors, including rotenone and paraquat, can lead to astrocyte senescence. Senescent astrocytes exhibit pro-inflammatory phenotypes similar to those of reactive astrocytes, while SASP secretion contributes to dopaminergic neuronal death in PD [91, 92]. Astrocyte aging is dependent on the activation of cGAS-STING signaling. The knockdown of cGAS in astrocytes results in a reduction in senescent astrocytes, reduced loss of dopaminergic neurons, and improved motor function [93]. Activated microglia can induce astrocyte reactivity by secreting high levels of IL-1α, TNFα, and C1q. Reactive astrocytes lose their capacity to promote neuronal synapse formation and phagocytose harmful substances, thus leading to neuronal and oligodendrocytes [94]. Flow cytometric analysis of the peripheral blood from patients with mild PD and age- and sex-matched controls revealed a significant reduction in the proportion of senescent CD8+T cells in patients with mild PD, while CD4+T cells did not exhibit significant changes. This contrasts with previous studies on patients with PD, where an increase in CD4+T cells, particularly effector CD4+T cells, was observed in the peripheral blood [95-97]. The varying manifestations of peripheral immunity in patients with PD across different studies may be associated with disease progression or drug intervention; however, these results collectively underscore the link between PD and peripheral immune activity, warranting further investigation. Future studies should investigate the intricate interplay between peripheral immunity and senescent cells in Parkinson's disease across the different stages of progression.

### 4.3 Multiple sclerosis

The onset and progression of multiple sclerosis are closely associated with age [98], with younger patients typically experiencing relapsing-remitting disease, while older patients are more prone to developing permanent disability [99]. Pathologically, this condition is characterized by the accumulation of CD8+ T and B cells in the limbic regions and the adjacent parenchyma of the central nervous system, accompanied by demyelination, axonal dysfunction, microglial activation, and astrocyte proliferation [100, 101]. Specifically, CD8+ T-cells, monocytes, and macrophages have all been observed to infiltrate the parenchyma [102]. Furthermore, research has shown that the development of multiple sclerosis is correlated with changes in aging peripheral immune cells. The telomere length of white blood cells in patients with multiple sclerosis is significantly shorter than that in healthy individuals [103]. Moreover, a correlation has been found between the degree of telomere shortening and severity of disability among these patients [104]. Additionally, bone marrow mesenchymal stem cells with multiple sclerosis exhibit shortened telomeres [105]. The shortening of telomeres in immune cells can further lead to DNA damage, thus promoting the premature senescence of T cells. The accumulation of senescent T cells thereby creates a pro-inflammatory environment that hinders myelin regeneration, contributing to the progression of multiple sclerosis [104]. Furthermore, it has been confirmed that senescent T cells predominantly comprise CD8+T cells, which are characterized by a decrease in the naïve T cell population, the accumulation of terminally differentiated memory T cells, and an increase in toxic cellular molecules [106-108]. A loss of axonal integrity and significant neurodegeneration have also been observed in mice exhibiting CD8+T cell senescence [109]. The pro-inflammatory environment within the central nervous system concurrently creates conducive conditions for the proliferation of pathogenic microglia [110], which, promote neuronal loss and dysfunction under pro-inflammatory states [111]. Pathogenic microglia have further been identified in demyelinating mouse models and patients with multiple sclerosis (MS) [112]. Indeed, the senescence of peripheral immune cells is particularly evident in MS, suggesting that MS is highly related to changes in the immune system.

### 4.4 Amyotrophic lateral sclerosis

The incidence of amyotrophic lateral sclerosis (ALS) rises with age, peaking after 60 years of age [113]. The key pathological mechanisms underlying ALS involve the progressive degeneration of both upper and lower motor neurons due to heightened central and peripheral inflammation [114]. Clinical data from a large cohort of patients with ALS has further shown that genetics plays a primary role in the development of ALS, with age serving as a significant factor in its onset and progression [115]. Many individuals with familial ALS carry mutations in the C9ORF72, leading to the accumulation of arginine-rich peptides that gradually impair nucleolar function and induce cell death. Furthermore, in addition to elucidating the potential mechanisms underlying ALS pathogenesis, these findings demonstrate that excessive expression of arginine-rich polypeptides in mice not only induces nucleolar stress, but also accelerates aging and reduces lifespan, thus highlighting the involvement of the nucleolus in individual senescence [116], suggesting a potential association between ALS and aging. Progress in genome sequencing has further led to the identification of numerous disease-causing genes in ALS, including SOD1, TARDBP, and C9orf72, many of which encode proteins that disrupt the immune system and exacerbate central nervous system inflammation [117-120]. Experiments using animal models created to study ALS pathogenic genes have revealed that the pathological mechanism of ALS involves not only motor neuron death but also abnormal responses from microglia, astrocytes, oligodendrocytes, and other neuronal populations [121]. Furthermore, it has been observed that peripheral immune cells play a role in the pathogenesis of ALS; indeed, peripheral blood mononuclear cells from ALS patients exhibit a pro-inflammatory phenotype [117]. A compromised blood-brain barrier is evident in both patients with ALS and mouse models, thereby facilitating the infiltration of activated monocytes and macrophages derived from the periphery into the central nervous system. This phenomenon enhances neuroinflammation and contributes to ALS [122].

### 4.5 Conclusions

Currently, research on degenerative diseases of the central nervous system is most advanced in AD, while investigations into PD, MS, and particularly ALS are relatively limited, possibly because of a lack of effective animal models. It has further been demonstrated that aging and immune cell activity may impact the function of the central nervous system. Animal models of aging may contribute to advancing the study of central nervous system degeneration by inducing senescence in specific cells within the central nervous system or immune system, thereby simulating the pathological mechanisms underlying related diseases and facilitating the exploration of new preventive and therapeutic strategies.

## 5. Central nervous system injury

Cellular senescence plays a crucial role in tissue repair and reconstruction following injury triggered by the SASP [123]. Indeed, the transient appearance of senescent fibroblasts and endothelial cells has been observed at the wound site upon skin injury in P16-3MR mice. Platelet-derived growth factors secreted by senescent cells further stimulate fibroblast differentiation into myofibroblasts, thereby facilitating wound contraction and tissue repair. However, failure to eliminate senescent cells may impede the tissue repair process [124]. Senescent cells directly promote tissue regeneration. For example, senescent stem cells in liver injury can promote the expression of stem cell genes in surrounding cells through the secretion of NF-κB, thereby contributing to liver regeneration following injury [125]. Central nervous system injury is usually accompanied by a vigorous inflammatory response, including the activation of central macrophages and microglia, the infiltration of peripheral monocytes and lymphocytes, and the accompanying release of inflammatory factors, which may be beneficial at some stage of the injury, The robust inflammatory response itself can lead to detrimental effects on the central nervous system [126]. Elevated levels of inflammatory mediators contribute to accelerated cellular senescence [127] and senescent cells serve as a potent source of inflammation [128]. Developing a strategy to harness the potential of senescent cells for tissue repair while mitigating further damage may thus represent a promising approach for the treatment of central nervous system injuries.

### 5.1 Brain injury

Brain injury is a form of trauma that results in the destruction or degeneration of brain cells, leading to trauma, stroke, and tumors. Brain injury can result in severe damage to neurological function, potentially leading to mortality or significantly impacting patient quality of life [129]. Large-scale observational cohort studies have demonstrated that brain injury can precipitate cognitive decline in young adults, while individuals with a history of brain injury are at an increased risk of developing AD; moreover, the frequency of injuries correlates positively with the likelihood of developing AS [130]. However, when comparing young and elderly patients with brain injury, a more pronounced acceleration of brain aging was observed in older individuals than in younger ones [131], suggesting greater vulnerability to brain injury in the elderly. This indicates a reciprocal relationship between aging and injury, with injury accelerating cellular senescence, and senescent cells exhibiting a reduced ability to withstand damaging stimuli. Following brain injury in rats, SA-β-gal activity increases in a time-dependent manner in a variety of brain regions, including the cortex, hippocampus, and thalamus. This change may underlie the disruptions in memory and impairments in motor, auditory, and visual functions [132]. Flow cytometry analysis of small mouse brain injury revealed a stronger potential for the appreciation of immune cells in old rats compared to in young rats, in addition to the increased infiltration of immune cells and secretion of brain inflammatory factors; however, microglia exhibit an aging phenotype. In contrast, microglia from young mice develop phenotypes resembling those of aged mice with increased expression of the senescent phenotype [133]. Senescent microglia are characterized by impaired phagocytosis and dysregulated inflammatory signaling, affecting their immune surveillance and clearance functions, and ultimately contributing to neurodegeneration and cognitive decline [134, 135]. Immune privilege in the normal brain is defined by a low number of T cells. However, brain aging disrupts this immune privilege, allowing for an increase in the infiltration of T cells into the brain. Brain injury exacerbates the loss of immune privilege, leading to increased T cell infiltration [136]. An increase in the number of T cells in the brain can further exacerbate neuroinflammation, potentially resulting in significant cognitive impairment [55].

### 5.2 Spinal Cord injury

Spinal cord injury is defined as a severe insult to the central nervous system [137, 138]. The imbalance caused by tissue, cellular, and molecular changes in the microenvironment at different time points and locations following spinal cord injury is a major contributor to impaired nerve regeneration and repair [139, 140]. Senescent cells can further promote a microenvironmental imbalance through the secretion of SASP, leading to increased pro-inflammatory mediators, neuronal dysfunction, and the polarization of microglia and astrocytes [141]. Neuronal senescence has also been observed following SCI in both zebrafish and mice. In the zebrafish spinal cord, an initial increase in senescent cells is observed, followed by a gradual decrease post-injury. Active elimination of senescent cells during the early stages of injury resulted in reduced nerve regeneration capacity, suggesting that the transient presence of senescent cells promotes nerve regeneration in a zebrafish model of spinal cord injury. In mice, an accumulation of senescent neurons and a decline in nerve regeneration ability were observed with prolonged injury times. However, treatment with drugs targeting senescent cells was shown to enhance nerve regeneration and reduce the inflammatory response [142]. This phenomenon was also evident in tissues of other organisms, such as salamanders, newborn mice, and zebrafish [143-145]. In salamanders, the long-term presence of senescent cells impedes tissue regeneration; however, macrophages play a crucial role in monitoring and eliminating senescent cells to promote tissue regeneration [145]. Macrophages have further been observed to play a crucial role in promoting tail fin regeneration in zebrafish. The depletion of macrophages through gene knockout resulted in a decreased regenerative capacity of zebrafish tail fins. In contrast, the depletion of macrophages in mammals affects wound bleeding, but does not affect their regenerative ability [146]. The differential impact of senescent neurons on spinal cord injury between zebrafish and mice is directly linked to the duration of senescence, which could potentially be attributed to variations in macrophage surveillance and the elimination of senescent cells. The fundamental reason underlying this phenomenon may be that zebrafish and mice have different genomes, as they are two distinct animal species. If this feature of zebrafish could be replicated in mammals, it may represent a promising therapeutic strategy. Furthermore, astrocyte senescence has been identified in aged mice following spinal cord injury, with these mice displaying increased susceptibility to aging and subsequent death post-injury. When P75 was knocked out to suppress senescence, the mortality rate of aged mice decreased. Furthermore, knockout of P75 in adult mice did not result in improved recovery of motor function, but did contribute to the restoration of microenvironmental homeostasis, indicating that spinal cord injury triggers astrocyte senescence and that senescent astrocytes exacerbate the surrounding environment [147]. Single-cell sequencing technology was employed to analyze immune cells following spinal cord injury in young and aged mice, revealing the significant infiltration of myeloid cells and the in situ activation of microglia in young mice, whereas the corresponding immune response was attenuated in aged mice, potentially accounting for their poorer recovery following SCI [148].

Senescent cells have further been observed in central nervous system injuries. Prolonged senescent cell activity leads to the establishment of a microenvironment rich in inflammatory mediators through the secretion of pro-inflammatory SASP. Under this environment, neuronal dysfunction or secondary injury may occur and immune cells become activated, exacerbating peripheral inflammation. However, the transient presence of senescent cells can promote tissue repair, partly because of the release of the SASP. The elimination of senescent cells by immune cells, including timely apoptosis to allow the removal of aging cells, presents a promising strategy for the treatment of central nervous system damage. Injury and cellular senescence predominantly exert deleterious effects on the central nervous system, and current studies largely support their mutual exacerbations. Consequently, attenuating the proportion of senescent cells during aging and inhibiting cell senescence following injury are pivotal strategies for preventing and treating injuries.

## 6. Neurological Neoplasms

Senescent cells play a crucial role in tumor initiation and progression. Importantly, senescence can impede tumor progression by suppressing cell proliferation. Indeed, various anticancer therapies, such as chemotherapy, radiotherapy, and targeted therapy, aim to induce senescence and eliminate cancer cells. However, these therapies may also simultaneously tumor occurrence, progression, and evasion. Indeed, senescent cells present in tumors can activate the immune system by recruiting T cells, macrophages, natural killer (NK) cells, neutrophils, and other immune cells through the secretion of SASP, or alteration of their surface antigens [149, 150]. Indeed, one study on lung cancer revealed that drug interventions aimed at inducing senescence in lung cancer cells resulted in enhanced activation and cytotoxicity of NK cells, thereby demonstrating the potential of inducing cancer cell senescence as a potential therapeutic target [151]. As such, current research indicates that the induction of tumor cell senescence to activate the immune system for the removal of aging tumor cells is a promising treatment strategy.

### 6.1 Immune profile in brain tumors

The immune environment in the central nervous system differs from that of other body systems, resulting in brain tumors having a unique immune profile [152]. For example, glioblastoma and other intracranial tumors have been observed to sequester T cells in a sphingoid 1-phosphate receptor 1 (S1PR1)-dependent manner, confining them to the bone marrow and impeding their surveillance and clearance of cancer cells [153]. Hence, immunotherapy regimens tailored specifically for central nervous system tumors may not yield the desired efficacy [154]. Compared to other tumor types, central nervous system tumors exhibit a significant reduction in infiltrating immune cells, particularly lymphocytes [155], and a lower number of specific T lymphocytes upon vaccination-induced T cell responses [156]. Indeed, research has shown that brain stromal cells secrete elevated levels of TGF and IL-10 inflammatory cytokines when stimulated by tumors, leading to the substantial inhibition of immune cell activation, and impacting the surveillance and clearance of tumor cells by immune cells [157, 158]. The immune activity of T cells in the brain is further modulated by the regulation of tryptophan and arginine levels. Due to the low concentration and limited availability of amino acids in the brain, resident cells are susceptible to fluctuations in amino acid levels [159]. Indoleamine 2,3-dioxygenase (IDO) secreted by neuroglioma cells can impede T cell function by depleting tryptophan in the microenvironment [160, 161]. Furthermore, certain immune cells also have the capacity to suppress T cell immune activity. Further, microglia and tumor-infiltrating bone marrow cells secrete a substantial amount of arginase to deplete arginine within the microenvironment, thereby inhibiting the proliferation and function of T cells [162] (149-151).

### 6.2 Senescence promotes tumorigenesis

Senescent cells have been identified in various central nervous system tumors, including craniopharyngiomas (CP), which are benign epithelial tumors located in the sellar region. These include ameloblastoma caused by the CTNNB1 mutation (ACP) and papillary cancer caused by the BRAF-V600E mutation (PCP) [163, 164]. Although senescent cells are not commonly observed in PCPS, they have been detected in human ACPs. Cell clusters of ACPs show no expression of Ki67, along with high expression of p21 and DNA damage markers such as γ-H2AX, as well as an activated DNA damage response indicated by phospho-DNAPKcs staining. Additionally, an enlargement of lysosomal compartments marked by GLB1 expression and activation of the NFκB pathway demonstrated through phosphorylated IκB staining were also noted [165]. Activation was also been reported [166]. To further investigate this, a genetically modified mouse model of ACP was established through the upregulation of β-catenin expression in pituitary SOX 2 ^positive^ stem cells during embryonic development and adulthood [167]. Senescent cell clusters were further identified in the mouse model, originating from β-catenin overexpressed SOX 2^positive^ stem cells; however, tumors arose from other cells lacking β-catenin expression, suggesting that β-catenin overexpressed SOX 2^+^ stem cells induce tumor formation through a cellular paracrine mechanism [168, 169]. This cell cluster is sensitive to senescence-clearing drugs and its capacity to drive tumor progression is diminished following senescence-clearing or SASP inhibition [170].

Low-grade glioma (LGG) is a slow-growing, non-invasive, benign brain tumor, characterized by a parenchymal mass that results in symptoms through the compression or invasion of surrounding tissues [171-173]. The majority of non-pilocytic astrocytomas (PAs) are associated with mutations in MAPK pathway activation [174], which can induce cancer gene aging, and have been observed in 90% of PA samples with enhanced beta-galactose-glucoside enzyme activity and increased p16 ^INK4a^ expression [175]. Patients with non-Pas, as well as mouse models, exhibit an increased SASP, which is linked to the prognosis of PAs [176]. Dexamethasone treatment inhibits SASP secretion by senescent cells and promotes their regrowth [177]. The deletion of P16 is infrequently observed in low-grade gliomas [178], whereas homozygous deletion of P16 is much more common in high-grade gliomas, such as pleomorphic xanthoastrocytoma and anaplastic astrocytoma with piloid features [179, 180]. This suggests that senescent cells promote the progression of low-grade gliomas into high-grade gliomas.

Glioblastoma multiforme (GBM) is one of the most prevalent primary brain tumors, characterized by high invasiveness, high recurrence rates, and low survival rates [181]. GBM typically involves the inhibition of P53 and RB pathways or the activation of the RAS, PI3K, and receptor tyrosine kinase pathways, which are commonly involved in oncogene-induced senescence [182]. Senescent cells have not been directly identified in GBM, however, treatment with Temozolomide in GBM cell lines has revealed the activation of senescent features, including increased P21 expression, DNA damage, activation of the NF-κB pathway and elevated SASP release such as IL-6 and IL-8 [183]. This phenomenon has also been observed in a mouse model of GBM [184]. Radiation exposure also induces aging of GBM cells, which exhibit more aggressive behavior [185]; however, the induced aging of GBM cells can be targeted with ABT-263, suggesting the potential for combined treatment strategies for GBM [186].

### 6.3 Senescence inhibits tumor progression

Medulloblastoma (MB), a tumor arising from distinct populations of neuronal progenitors, is the most prevalent high-grade pediatric brain tumor in children, and is classified into four major subgroups: WNT, SHH, Group 3, and Group 4 [187, 188]. In MB model mice, tumor lesions were found to contain senescent cells expressing p16, INK4a, p21, and Cip1, but senescent cells but were not detected in late-stage tumors [189]. This suggests that the elimination of senescent cells forms one of the bases of tumor progression [190].

Diffuse midline glioma (DMG), an incurable grade IV pediatric tumor, is characterized by mutations in histone-coding genes, specifically in the histone H3 gene H3F3A (encoding H3.3) or the related HIST1H3B (encoding H3.1), and is often associated with TP53 deletion [191]. The associated low levels of aging markers may be linked to the inhibition of the CDKN2A gene [192]; however, the observation of P16 positivity in tumor cells indicates its potential to promote tumorigenesis [193].

In central nervous system tumors, senescent cells serve as both initiation and progression factors during the early stages of cancer. Senescence markers can be used as indicators of cancer detection and standards for determining prognosis. However, the induction of senescence in tumor cells can also inhibit their proliferation. In advanced or aggressive tumors, in which senescent cells are rarely found, it is evident that these cells impose certain restrictions on tumor development. Furthermore, senescent cells can enhance immune activity within the central nervous system by activating immune cells, leading to increased surveillance of tumor cells and potentially serving as a novel treatment strategy.

**Table 2:** **Targeted senescence treatment**. Here, we present some representative approaches to alleviate aging using different anti-aging strategies in recent years.

	Method	Species	Condition	Outcome	Reference
Medicine	D+Q	Mice	Alzheimer’s Disease	Amyloid plaque degradation, cognitive function improved	Choi et al., 2023
	D+Q	Human	Alzheimer’s Disease	Amyloid and inflammatory factor drop	Gonzales et al., 2023
	D+Q ABT-737	Human	SARS-CoV-2 infection	The survival rate of dopaminergic neurons increased; the age-related proteins decreased	Aguado et al., 2023
	ABT-263	Mice	Spinal cord injury	Senescent cells decreased and movement improved	Da Silva-Álvarez et al.,2020
	ABT-263	Zebrafish	Spinal cord injury	Senescent cells decreased; tissue repair weakened	Da Silva-Álvarez et al.,2020
	rapamycin	Mice	Neural tube defect	The incidence of neural tube defects decreased	C. Xu et al., 2021
	Trametinib palbociclib	Mice	Lung cancer	Induce senescence of cancer cells and enhance NK cell killing of cancer cells	Ruscetti et al., 2018
	Ascorbic acid	Monkey	Aging	Senescent cells decreased and movement improved	Sun et al., 2023
Immunotherapy	CAR-T	Human	Glioblastoma	The risk of tumor escape was reduced	Pellegatta et al., 2018
	CAR-T	Mice	Aging	Exercise and metabolic function improved	Amor et al., 2024
	CAR-T	Mice	Aging/Radiate	Aging function is improved,age-related diseases are reduced	D. Yang et al., 2023
	Vaccine	Mice	Aging	Senescent cells decreased and live longer	M. Xu et al., 2018
**Systemic anti-aging**	Transfusion of young blood	Mice	Aging	Cognitive function improved, memory and learning ability enhanced	Villeda et al., 2014
	Transfusion of human cord blood	Mice	Aging	Cognitive function improved, memory and learning ability enhanced	Castellano et al., 2017
	Transfusion of young cerebrospinal fluid	Mice	Aging	The proliferation and differentiation of hippocampal oligodendrocytes were enhanced	Iram et al., 2022
	Young bone marrow transplantation	Mice	Aging	Activity levels, learning ability, spatial memory and overall brain vitality improved	Das et al., 2019

## 7. Senescence as a treatment target

Owing to the significant roles of senescent cells under both physiological and pathological conditions, the targeting these cells has emerged as a crucial therapeutic strategy [194-196]. The application of these treatment strategies to central nervous system diseases also holds promise for enhancing treatment efficacy. This article presents advanced and well-established treatments for senescent cells, offering insights into potential approaches for treating central nervous system diseases (Table 2).

### 7.1 Senolytics

Senolytics specifically target and eliminate senescent cells. Although most factors that cause cellular senescence also induce apoptosis, the resistance of senescent cells to apoptosis allows them to persist in a senescent state. This heightened sensitivity of senescent cells to inhibitors of apoptotic proteins has led to the discovery of potential senolytic agents, including a combination of multiple tyrosine kinase inhibitors (D) and the flavonol quercetin (Q). Subsequently, inhibitors targeting the anti-apoptotic protein BCL-2, such as ABT-263 and ABT-737, were also identified using similar approaches. In the context of research on central nervous system diseases, senolytic treatment comprising D+Q has been shown to effectively eliminate aging brain amyloid plaques surrounding microglia in a mouse model of AD. This treatment also enhanced the phagocytic activity of microglia, promoted the degradation of amyloid plaques, and improved cognitive function in mice [197]. Furthermore, an increase in drug concentration was observed in the cerebrospinal fluid of patients in clinical trials investigating the utility of D+Q for AD treatment. Importantly, this treatment was found to be safe and feasible, with no significant changes detected in memory or brain imaging; however, a reduction in brain amyloid and inflammatory factor levels was noted following treatment. Overall, these findings provide evidence supporting the safety and tolerability of this therapeutic approach [198]. During the coronavirus disease (COVID-19) pandemic, it was discovered that SARS-CoV-2 infection could induce cellular senescence in brain organoids. Treatment with senolytics to selectively eliminate senescent cells in the brains of mice with SARS-CoV-2 infection resulted in improvements in clinical manifestations and survival rates, reduced viral load in the brain, and enhanced survival of dopaminergic neurons. This treatment further led to decreased astrocyte proliferation, reduced aging-related infections, and downregulated expression of SASP-related genes [199]. Additionally, emerging drugs such as heat shock protein inhibitors [200], cardiac glycosides [201], and P53 and MDM2 inhibitors [202] have all shown promise as potential senolytics.

### 7.2 Immunotherapy

Senescent cells exhibit distinct protein phenotypes that make them susceptible to targeted elimination by immune cells, or specific surface antigens that can be recognized by antibodies. For example, chimeric antigen receptor T (CAR-T) cell therapy can redirect T cells to selectively eliminate cells expressing the target antigen, particularly when the antigen is expressed differentially in diseased tissues compared to normal tissues [203]. The application of CAR T-cell therapy in cancer treatment has been well established, with significant efficacy achieved by targeting CD 19 in patients with refractory B-cell malignancies [204]with control of the growth of pancreatic ductal adenocarcinoma, ovarian cancer, and neuroblastoma further achieved by targeting B7-H3, both in vitro and in vivo [205]. Furthermore, targeting chondroitin sulfate proteoglycan 4 (CSPG4) in glioblastoma has shown promise in reducing the risk of tumor cell escape when the targeted antigen is heterogeneously expressed in tumor cells within the central nervous system [206]. The optimal target antigen for CAR T cells should be specifically expressed in the target cell, while avoiding expression in other tissues. Through genomic analysis of three independent aging models, the urokinase-type plasminogen activator receptor (uPAR) was identified as a suitable target for CAR T-cell therapy, aimed at eliminating senescent cells. In vivo and in vitro validation studies have further demonstrated that CAR T cells targeting uPAR can effectively eliminate senescent cells [207]. Subsequent studies have also shown that the specific depletion of senescent cells using uPAR-directed CAR T cells improved exercise capacity and metabolic dysfunction, such as glucose tolerance, in aged mice, as well as those on a high-fat diet, without causing tissue damage or toxic effects [208]. Additionally, NKG2D-targeted CAR-T cells selectively eliminate senescent cells, enhance physical performance, and reduce the incidence of age-related diseases in both aged and irradiated mice [209].

Antibody-dependent cytotoxicity is another form of immunotherapy in which antibodies guide immune cells to specifically target and eliminate senescent cells. Senescent cells express dipeptidyl peptidase 4 (DDP4) on their surface; as such, anti-DDP4 antibodies can label these cells and direct NK cells to selectively eliminate DDP4-positive senescent cells [210]. Additionally, antibody-drug conjugates (ADCs) have demonstrated efficacy in eliminating senescent cells. For example, a B2 M IgG1 monoclonal antibody conjugated with bectinomycin, an irreversible DNA alkylating agent, can recognize and bind to extracellular B2 M on the surface of SnCs, leading to selective cell death of SNCS following fractionation and release [211]. The utilization of a senescence-eliminating GPNMB vaccine in mice has further demonstrated a reduction in the number of senescent cells and an extension of lifespan, suggesting that anti-aging vaccines hold promise as immunotherapies. In comparison to senolytics, immunotherapeutics exhibit stronger targeting capabilities and fewer side effects [212]. Indeed, recent research discovered that the administration of BCG to aged mice can induce the training of microglia in the central nervous system, enhancing their phagocytic function and facilitating the clearance of lipid fragments from the myelin sheath, ultimately promoting myelin regeneration [213]. However, this strategy has not yet been fully developed for clinical treatment and requires further exploration and development [214].

### 7.3 Systemic anti-aging

Systemic antiaging interventions are promising strategies for mitigating the onset of age-related symptoms and diseases. Plasma proteomic analysis of over five thousand individuals has shown that the expression levels of plasma proteins can serve as indicators of aging across 11 organs, tissues, and systems in the human body, underscoring the close relationship between blood composition and the overall aging process [215]. Notably, infusion of young mouse blood into aged mice resulted in increased expression of vascular cell adhesion molecule-1 (VCAM-1) in endothelial cells, reduced activity of neural progenitor cells destined to become neurons, and heightened microglial activity in brain immune cells, ultimately leading to improved cognitive function and enhanced memory and learning abilities in aged mice [216]. Experiments involving the injection of human umbilical cord blood plasma, young adult plasma, and old adult plasma into aged mice further demonstrated that the injection of human umbilical cord blood plasma and young adult plasma enhanced memory and learning ability in aged mice and improved hippocampal function [217]. Young blood possesses anti-aging properties, whereas older blood can accelerate aging. Surgical ligation experiments involving the sharing of blood, organs, and the environment between young and old mice have shown that old mouse blood induces senescence in the cells and tissues of young animals after a single exchange. However, this effect is mitigated if the old animals are treated with anti-aging drugs prior to the exchange. This suggests that the pro-aging effects of old blood in young mice could be attenuated by the application of appropriate interventions [214]. The anti-aging effect of young blood on the central nervous system may be influenced by the presence of blood-brain and blood-spinal cord barriers [218]. Indeed, the cerebrospinal fluid is closely associated with the health of the central nervous system, and its protein composition changes with age [219], characterized primarily by an increase in pro-inflammatory molecules [220] and a decrease in growth factors [221]. Indeed, studies investigating the effects of the injection of CSF from young mice into aged mice revealed that young cerebrospinal fluid enhanced the proliferation and differentiation of oligodendrocytes in the hippocampi of aged mice. This phenomenon has also been observed in primary cultured oligodendrocytes in vitro. Fibroblast growth factor 17 (Fgf 17) has further emerged as a key target, being more abundant in young cerebrospinal fluid [222]. Aging of hematopoietic stem cells (HSC) also has a profound impact on systemic aging. The functionality of HSCs undergoes significant changes with age, and is characterized by a compromised regenerative capacity, altered differentiation propensity, and heightened susceptibility to malignancy [223, 224]. Transplantation of bone marrow from young mice into old mice resulted in improved activity levels, learning ability, spatial memory, and overall brain vitality compared with old mice transplanted with bone marrow from their peers [225]. Conversely, the transplantation of aged mouse bone marrow into young mice has been shown to lead to impaired regeneration and persistently-biased differentiation within the downstream progenitor cells of HSCs, resulting in negligible functional recovery. It was further discovered that the DNA methylation profile of HSCs was altered in aged mice, and that this change could not be reversed by the young bone marrow microenvironment, indicating a potential cause of aging-induced dysfunction of HSCs [226]. In addition to replacing aged HSCs with their young counterparts, the rejuvenation of HSCs could also be achieved through the depletion of senescent HSCs. In one study, depletion of myeloid HSCs using antibodies revitalized the immune system of elderly mice, leading to an increase in common lymphocyte progenitors, naïve T cells, and B cells, while reducing markers associated with age-related immune decline [227]. Nevertheless, despite the above research, there is still significant progress to be made in systemic anti-aging research, which holds promise for advancing the diagnosis, treatment, and prevention of central nervous system age-related diseases, as well as all other age-related conditions, and potentially extending the lifespan.

Senescent cells play an increasingly pivotal role in the investigation of various diseases and injuries affecting the central nervous system, with a growing emphasis being on the therapeutic strategies targeting these cells. The application of established therapeutic approaches to other diseases of the central nervous system, as well as the development of novel therapeutic strategies, presents both opportunities and challenges in this field. For example, the use of drugs to remove senescent cells or actively induce cell senescence to treat diseases has been relatively mature in animal experiments, and the primary challenge is therefore to confirm whether they are effective and safe from a clinical perspective. Immunotherapy and systemic therapy are still far from being applied at the clinical level; however, research can still help to allow a deeper understanding of cellular aging in the nervous and immune systems, and for promoting the development of relevant therapeutic strategies.

## 8. Discussion

Substantial evidence has indicated a pivotal role of senescent cells in the pathogenesis of central nervous system development, aging, degenerative diseases, injury, and cancer, with numerous therapeutic interventions targeting senescent cells having demonstrated promising clinical and experimental outcomes. However, the precise mechanisms underlying central nervous system aging remain elusive, impeding the development of anti-aging therapies for neurological disorders.

Senescent cells are typically multifaceted. Senescent cell proliferation stagnation can prevent aberrant cell proliferation, as well as facilitating normal embryonic development and cellular renewal during embryogenesis and cancer cell growth. The dysfunction of senescent cells is a major contributor to neurodegeneration, such as the impaired functionality of senescent neurons in Alzheimer's disease. Another hallmark of senescent cells is the secretion of SASP, which primarily relies on their functional components. For instance, TGFβ1 within SASP can promote fibrosis and restrict inflammatory cell dissemination in spinal cord injury; however, it can also induce cellular senescence and impact normal tissue function. Untimely cellular senescence is also associated with embryonic malformations and degenerative diseases, impeded tissue repair, and the promotion of cancer cell metastasis. However, the precise timing of senescent cell involvement in various physiological and pathological mechanisms of the central nervous system remains poorly understood. Despite the promising results from numerous targeted therapies aimed at eliminating senescent cells, the optimal timing for their depletion remains uncertain. For example, contrasting roles for senescent cells have been observed in zebrafish and mice with spinal cord injuries. The underlying biological mechanisms responsible for this heterogeneity among senescent cells are not yet well understood, and whether it stems from population differences between species, or involves molecular switches that regulate their biological functions, remains unknown.

The secretion of the SASP by senescent cells warrants further investigation. Although many cellular aging processes have been attributed to the release of SASP, direct evidence to support these hypotheses is lacking. The composition of the SASP produced by different cell types varies significantly, particularly in the context of nervous system aging. Given the complexity of the cellular composition in the nervous system, more precise models involving aged cells or animals are required to identify the specific components of the SASP. Furthermore, advances in single-cell sequencing technology have facilitated the establishment of distinct SASP profiles in various senescent cells. By precisely regulating the composition of the SASP, its beneficial effects can be maximized while minimizing potential harm.

Until recently, studies on the effects of cellular senescence on the central nervous system and immune system were predominantly conducted in isolation. However, recent research has established that cell senescence can serve as a crucial link between the central nervous system and immune system. Senescent neurons or glial cells in the central nervous system attract immune cells that either directly eliminate senescent cells or induce apoptosis. However, if immune cells undergo senescence, their clearance ability is compromised, leading to the accumulation of senescent cells within the central nervous system, which can promote disease progression. The preservation of youthful characteristics is thus essential for maintaining the optimal functioning of both the nervous and immune systems; thus, understanding how aging influences bone marrow hematopoietic stem cells, which are fundamental components of the immune system, along with studying their interaction with the bone marrow microenvironment, is imperative. Age-related changes have also emerged as key factors influencing this interplay, making the exploration of strategies that delay aging-associated decline in immune function vital. Additionally, excessive activity of immune cells during central nervous system diseases or injuries may trigger neuronal or glial cell aging within the CNS, thereby exacerbating dysfunction within this critical system. Consequently, the control of immune cell activity is of paramount importance.

To investigate the specific mechanisms underlying aging in the central nervous system and to facilitate the application of anti-aging therapies for central nervous system diseases, it will be imperative to enhance research on age-related immune activities. Therapies targeting cellular aging in the nervous and immune systems or improvements in systemic aging throughout the body are potential options for treating age-related diseases. In addition, antiaging treatments can delay the decline in tissue function, prevent disease, and prolong life. This decline in nervous system function seriously affects people's cognitive and motor functions, and a delay in nervous system aging will greatly improve the quality of life of the elderly. Overall, a growing body of research has been conducted to elucidate the interplay between aging and immune function in various central nervous system disorders. Although most of these investigations are still far from being translated into clinical practice, antiaging interventions nevertheless have shown immense potential.

## Data Availability

No data was used for the research described in the article.
